# Comparison of three clinical scoring tools for bronchiolitis to predict the need for respiratory support and length of stay in neonates and infants up to three months of age

**DOI:** 10.3389/fped.2023.1040354

**Published:** 2023-02-17

**Authors:** Domenico Umberto De Rose, Chiara Maddaloni, Ludovica Martini, Annabella Braguglia, Andrea Dotta, Cinzia Auriti

**Affiliations:** ^1^ Neonatal Intensive Care Unit, Bambino Gesù Children's Hospital IRCCS, Rome, Italy; ^2^ Neonatal Sub-Intensive Care Unit and Follow-up, Bambino Gesù Children's Hospital IRCCS, Rome, Italy

**Keywords:** bronchiolitis, newborns, RSV, rhinovirus, viruses, influenza, respiratory infections, clinical scoring tools for bronchiolitis

## Abstract

**Background:**

Bronchiolitis severity can be assessed using different clinical scores. Some of the most used are the Wang Bronchiolitis Severity Score (WBSS), the Kristjansson Respiratory Score (KRS), and the Global Respiratory Severity Score (GRSS), calculated on the vital parameters and the clinical conditions.

**Objective:**

To assess which of the three clinical scores better predicts the need for respiratory support and length of hospital stay in neonates and infants younger than three months, admitted to neonatal units for bronchiolitis.

**Methods:**

Neonates and infants younger than three months admitted to neonatal units from October 2021 to March 2022 were included in this retrospective study. The scores were calculated in all patients soon after admission.

**Results:**

Ninety-six patients (of whom 61 neonates) admitted for bronchiolitis were included in the analysis. Median WBSS at admission was 4.00 (interquartile range, IQR 3.00–6.00), median KRS was 4.00 (IQR 3.00–5.00), and median GRSS 4.90 (IQR 3.89–6.10). We found significant differences in all three scores between infants who needed respiratory support (72.9%) and those who did not (27.1%) (*p* < 0.001). A value >3 for WBSS, > 3 for KRS, and >3.8 for GRSS were accurate in predicting the need for respiratory support, with a sensitivity of 85.71%, 75.71%, and 93.75% and a specificity of 80.77%, 92.31%, and 88.24%, respectively. The three infants who required mechanical ventilation had a median WBSS of 6.00 (IQR 5.00–6.50), a KRS of 7.00 (IQR 5.00–7.00), and a GRSS of 7.38 (IQR 5.59–7.39). The median length of stay was 5 days (IQR 4–8). All three scores were significantly correlated with the length of stay, although with a low correlation coefficient: WBSS with an r^2^ of 0.139 (*p* < 0.001), KRS with an r^2^ of 0.137 (*p* < 0.001), and GRSS with an r^2^ of 0.170 (*p* < 0.001).

**Conclusion:**

Clinical scores WBSS, KRS, and GRSS calculated on admission accurately predict the need for respiratory support and the length of hospital stay in neonates and infants younger than three months with bronchiolitis. The GRSS score seems to better discriminate the need for respiratory support than the others.

## Introduction

Bronchiolitis is a common lower respiratory tract infection affecting children younger than 24 months, usually from October-November to March-April in Italy. During the last two years COVID-19 pandemic altered this usual trend in Italy as in other countries (1). It occurs with rhinitis, persistent cough, and respiratory distress in the presence of wheezing or crackles to chest auscultation. Neonates and infants younger than three months are at risk for severe bronchiolitis with respiratory failure and prolonged hospitalization than older children (2). Currently, the mainstay of therapy is supportive care, including primarily respiratory support and intravenous hydration for more severe cases (2), without solid evidence about interventional therapy (3). The literature reports several clinical severity scores to assess the severity of bronchiolitis, such as the Wang Bronchiolitis Severity Score (WBSS) (4), the Kristjansson Respiratory Score (KRS) (5), the Tal score (TS) and the modified-Tal score (mTS) (6), and the Respiratory Distress Assessment Instrument (RDAI) (7). Recently, Rodriguez-Martinez et al. summarized in a meta-analysis up to 32 tools evaluating the severity of bronchiolitis, calculated on the vital parameters and the clinical conditions on admission (8). However, there is no consensus on which score is most useful in judging the severity of the infection. In particular, the Wang Bronchiolitis Severity Score (WBSS) has been applied to low respiratory tract infections for the last 30 years (4). The Kristjansson Respiratory Score (KRS), which is similar to WBSS, has shown higher inter-rater reliability and seems more adequate for rapid use in the Emergency Department (ED) (5). Both the scores are based on clinical signs and symptoms and are easy to use. The Global Respiratory Severity Score (GRSS) was developed in 2017 as a research tool to evaluate infants with RSV infection. It is an algorithm-based score including age-specific respiratory and general parameters (9). Recently, Kubota et al. described the GRSS score as useful in determining the need for respiratory support in infants aged under 10 months with an RSV infection (10).

Considering the usefulness of a clinical screening in the emergency room, to evaluate infants with bronchiolitis at great risk of respiratory failure and that none of the scores described is part of the clinical practice among neonatologists still now, we carried out a retrospective study to compare the accuracy of three clinical scores (WBSS, KRS, and GRSS) in discriminating the need for respiratory support (high-flow nasal cannula, nasal continuous positive airway pressure or mechanical ventilation) in small infants with bronchiolitis (neonates and infants younger than three months of age). We also assessed the correlation between the scores and the length of hospital stay in this category.

## Methods

### Study design

We retrospectively collected data (gender, gestational age, birthweight, age and weight at admission, need for non-invasive or invasive respiratory support, need for intravenous infusion, need for enteral fasting) from the medical records of neonates and infants aged <3 months, admitted to the Neonatal Intensive Care Unit and Neonatal Sub-Intensive Care Unit of our hospital for bronchiolitis from October 2021 to March 2022. We calculated clinical severity scores (WBSS, KRS, and eventually GRSS for patients with RSV infection only) on admission clinical data. Table 1 shows the parameters of each score. The WBSS consists of four items (respiratory rate, general appearance, wheezing, retractions), each ranging from 0 to 3, except for the general condition, which is scored only 0 and 3, with a total from 0 to 12. The KRS is based on five signs (respiratory rate, general appearance, wheezing, retractions and skin color), each from 0 to 2, with a total from 0 to 10. The GRSS is calculated entering ten parameters (age, oxygen saturation, respiratory rate, general appearance, wheezing, rhales/ronchi, retractions, skin color, lethargy, and poor air movement) in an interactive tool (available at: https://rprc.urmc.rochester.edu/app/AsPIRES/RSV-GRSS/).

**Table 1:** Parameters of each scoring system.

	Wang Bronchiolitis Severity Score (WBSS) (4)	Kristjansson Respiratory Score (KRS) (5)	Global Respiratory Severity Score (GRSS) (9)
*Age (months)*	<24 months	NA	<10 months
*Oxygen saturation (%)*	NA	NA	67–100
*Respiratory rate*	<30: 0 30–45: 1 46–60: 2 >60: 3	<40: 0 40–60: 1 >60: 2	30–123
*General appearance*	Normal: 0 Abnormal: 3 (irritable, lethargic, poor feeding)	Normal: 0 Moderately affected: 1 Severely affected: 2	Well, mild, moderate, severe, NA
*Wheezing* *Rhales/rhonchi*	None: 0 Terminal expiration or only with stethoscope:1 Entire expiration or audible on expiration without stethoscope:2 Inspiration and expiration, without a stethoscope: 3	None: 1 Wheeze +/- ronchi or rales: 1 Severe wheeze +/- ronchi or rales: 2	Yes, No, NA Yes, No, NA
*Retractions*	None:0 Intercostal: 1 Tracheosternal:2 Severe with nasal flaring: 3	None:0 Moderate (costodiaphragmatic):1 Severe (as in 1 plus rib and jugular): 2	Yes, No, NA
*Skin color*	NA	Normal:0 Pallor:1 Cyanosis:2	Cyanosis (Yes, No, NA)
*Lethargy present*	NA	NA	Yes, No, NA
*Poor air movement*	NA	NA	Yes, No, NA

NA, not applicable. WBSS ranges from 0 to 12. KRS ranges from 0 to 10. GRSS scoring is according to the algorithm.

We excluded infants hospitalized only for apnoea and with any high-risk conditions for respiratory failure (congenital heart disease, neurologic disorders, and immunodeficiency), those qualified for palivizumab prophylaxis, and those with incomplete clinical data.

Infants were discharged 24 h after they no longer needed respiratory support and they achieved full enteral feeding again, and they no longer needed intravenous infusion.

The primary outcome was the need for respiratory support (either high-flow nasal cannula, nasal continuous positive airway pressure, or mechanical ventilation).

The secondary outcome was the length of hospital stay (days).

### Management of bronchiolitis during hospitalization

Nebulized 3% hypertonic saline solution, superficial nasal aspiration, and intravenous fluid therapy are used in our units, according to the latest guidelines and recent data from the literature (2, 11–14). Intravenous fluid therapy is rapidly decreased when the clinical conditions are adequate to provide enteral feeding. Patients with persistent saturation levels below 92% and signs of respiratory distress (tachypnoea, chest retractions, etc.) or respiratory acidaemia on the venous blood gas analysis undergo high-flow nasal cannula (HFNC) as primary respiratory support: we provide a flow rate of 2 liters/minute per kilogram of body weight, starting with 4 liters/minute up to 10 liters/minute. We use nasal continuous positive airway pressure (nCPAP) or mechanical ventilation as rescue therapy for those patients with clinical deterioration. In the case of nCPAP, positive end-expiratory pressure (PEEP) is set between 5 and 7 cmH20.

### Microbiology testing

All patients enrolled had been studied with nasopharyngeal swabs for the identification of respiratory viruses (Influenza virus, Respiratory syncytial virus, Adenovirus, Enterovirus, Parainfluenza virus, Metapneumovirus, Bocavirus, Rhinovirus, and Coronaviruses, including NL63/229E/OC43 and SARS-CoV-2) was done on nasopharyngeal aspirates by the multiplex real-time polymerase chain reaction (RT-PCR) “AllplexTM Respiratory Panel Assays” on All-in-One Platform (Seegene, Korea), as previously described (15).

### Ethical statement

The authors assert that all procedures of the study comply with the ethical standards of the institutional and national research committee and with the 1,964 Helsinki Declaration and its later amendments or comparable ethical standards (16). Personal data were restricted to essential information and were treated in order to guarantee the respect of the privacy of the involved patients, as specifically stated by Italian Law D. Lgs. n.196 of 2003 about personal data protection. Written informed consent was not required, as the study is retrospective with no patient-identifiable information. Despite this, our Scientific Directorate validated the study before the submission to the journal, as in our hospital all studies performed have to be approved by this office.

### Statistical analysis

Data are presented as numbers and percentages for categorical variables for statistical analyses. Continuous variables are expressed as mean ± standard deviation (SD) if normally distributed or as median and interquartile range (IQR) if normality could not be accepted. Data distribution was evaluated by the Shapiro–Wilk test. Comparisons between groups were made with Fisher test, t-test or Mann-Whitney test as appropriate (i.e., infants who needed respiratory support vs. those who did not, infants with RSV-bronchiolitis vs. infants with other viruses, and infants who required up to CPAP vs. infants managed only with HFNC).

We calculated the accuracy (sensitivity, specificity, positive predictive value, and negative predictive value) of three different clinical scores in discriminating the need for respiratory support with bronchiolitis. By the receiver operating characteristic (ROC) analysis, the area under the ROC curve (AUC) and the Youden's index (best cut-off point) were used to evaluate the ability of the single score to predict the need for respiratory support. Moreover, we calculated the correlation between scores and the length of hospital stay.

The three clinical bronchiolitis scores were set as independent variables and the clinical variables as dependent variables in linear regression models. The proportion of patients hospitalized due to severe bronchiolitis requiring supplemental oxygen was considered pre-test probability in estimating post-test probability. A *p*-value <0.05 was considered statistically significant. Data were analyzed with the MedCalc Software package for Windows, release 12.7 (MedCalc Software, Belgium).

## Results

From 1st October 2021 to 31st March 2022, we admitted 101 neonates and infants with acute bronchiolitis. Five infants were excluded because of incomplete data (lack of information about the worst oxygen saturation and respiratory rates in clinical records). Therefore, we included 96 infants (Table 2). Sixty-one (63.5%) were neonates, whereas 35 (36.5%) were within three months of life. Seven patients (7.3%) were born preterm (range: 32–36 weeks of gestational age). Among 71 infants who required respiratory support, three infants/71 (4.2%) received HFNC, nCPAP, and mechanical ventilation; nine infants/71 received (12.7%) HFNC and nCPAP; fifty-nine infants/71 (83.1%) received only HFNC. The Table 3 shows viruses causing bronchiolitis in our patients.

**Table 2:** Clinical characteristics and procedures of included patients with acute bronchiolitis.

	Patients (*n* = 97)
***Clinical characteristics***	
Males, *n* (%)	42 (43.3%)
Median gestational age, weeks (IQR)	39 (38–40)
Median birthweight, grams (IQR)	3,225 (2935–3510)
Median age at admission, days	24 (16–35)
Median weight at admission, grams (IQR)	3,530 (3180–4054)
***Procedures***	
Need for the high-flow nasal cannula (HFNC), *n* (%)	71 (73.2%)
Need for supplemental oxygen >21%, *n* (%)	43 (44.3%)
Need for nasal continuous positive airway pressure (nCPAP), *n* (%)	12 (12.4%)
Need for mechanical ventilation, *n* (%)	3 (3.1%)
Need for intravenous infusion, *n* (%)	87 (89.7%)
Need for enteral fasting, *n* (%)	9 (9.3%)

**Table 3:** Identified microorganisms causing bronchiolitis.

	Patients (*n* = 97)
Respiratory syncytial virus	80 (82.5%)
Rhinovirus	7 (7.2%)
Metapneumovirus	6 (6.2%)
Parainfluenza virus	3 (3.1%)

In all included infants, the median WBSS at admission was 4.00 (IQR 3.00–6.00), and the median KRS was 4.00 (IQR 3.00–5.00). In RSV infants, the median GRSS was 4.90 (IQR 3.89–6.10). The Table 4 shows the significant differences in the score calculation in patients needing or not respiratory support.

**Table 4:** Differences in bronchiolitis scores (WBSS, KRS, GRSS) between infants who needed respiratory support (HFNC, nCPAP or mechanical ventilation) or not.

	Respiratory support (*n* = 71)	No respiratory support (*n* = 26)	*p*-value
**Wang respiratory score (WBSS)**	5.00 (IQR 4.00–6.00)	2.50 (IQR 2.00–3.00)	<0.001
**Kristjansson respiratory score (KRS)**	4.00 (IQR 4.00–6.00)	2.00 (IQR 2.00–3.00)	<0.001
**Global Respiratory Severity Score (GRSS) for RSV infants**	5.30 (IQR 4.32–6.26)	3.13 (IQR 2.50–3.50)	<0.001

Infants who needed respiratory support had a lower age (but not significantly different) at admission (median 23.5 days, IQR 15.0–34.0 vs. median 25.5 days, IQR 18.0–36.0) (*p* = 0.322). Weight at admission was similar between the two groups, with a median of 3,490 grams (IQR 3135–3940) vs. a median of 3590 grams (IQR 3320–4100) (*p* = 0.288). The length of stay was significantly lower in the group of infants who do not require respiratory support (median 3.0 days; IQR 1.0–4.0) compared to more seriously ill infants (median 6.0 days; IQR 5.0–8.0) (*p* < 0,001).

Infants with RSV-bronchiolitis had a significantly higher WBSS score (median 4.50; IQR 3.75–6.00) rather than infants with other viruses (median 3.00; IQR 2.00–4.00) (*p* < 0.001). Similarly, infants with RSV-bronchiolitis had a significantly higher KRS score (median 4.00; IQR 3.00–5.00) rather than infants with other viruses (median 3.00; IQR 2.00–3.00) (*p* < 0.001).

**Figure 1:**
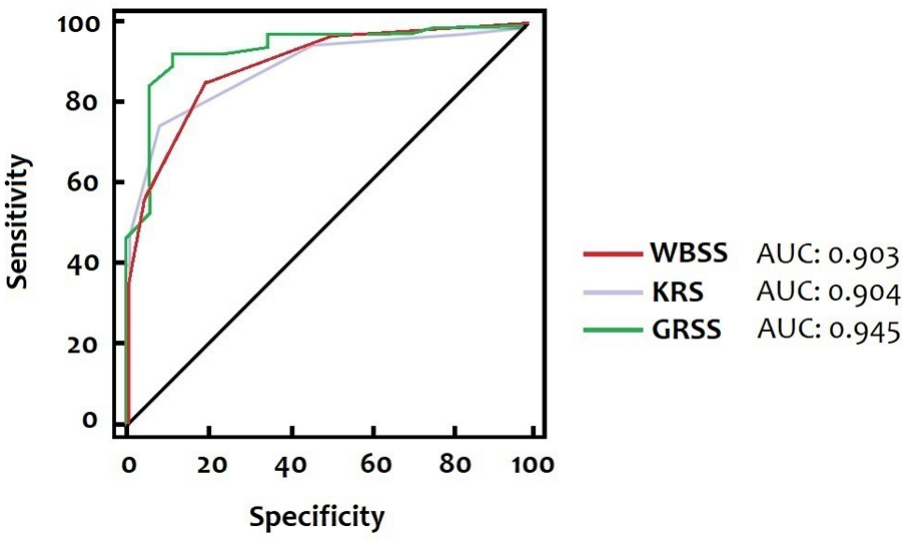
Receiver-operating characteristic curves of three different scores (WBSS, KRS, GRSS). The AUC for the WBSS, the KRS, and the GRSS is 0.903, 0.904, and 0.945, respectively, corresponding to cut-off values of >3, > 3, and > 3.8.

Table 5 shows the sensitivity, specificity, positive predictive value, and negative predictive value of the three different scores at the optimal cut-off to predict the need for respiratory support, identified by the ROC curve (Figure 1).

**Table 5:** Sensitivity, specificity, and positive and negative predictive value for the need of respiratory support (HFNC, nCPAP or mechanical ventilation) of the three bronchiolitis scores (WBSS, KRS, GRSS) at the optimal cut-off point.

	Cut-off	Sensitivity (95% CI)	Specificity (95% CI)	Positive Predictive Value (PPV) (95% CI)	Negative Predictive Value (NPV) (95% CI)	Post-Test Probability
**Wang respiratory score (WBSS)**	> 3	85.71 (75.3–92.9)	80.77 (60.6–93.4)	92.3 (92-9–97.4)	67.7 (48.6–83.3)	78%
**Kristjansson respiratory score (KRS)**	> 3	75.71 (64.0–85.2)	92.31 (74.8–98.8)	96.4 (87.4–99.5)	58.5 (42.1–73.7)	89%
**Global Respiratory Severity Score (GRSS)**	> 3.8	93.75 (84.7 -98.2)	88.24 (63.5–98.2)	96.8 (88.7–99.5)	78.9 (53.6–94.1)	87%

**Figure 2:**
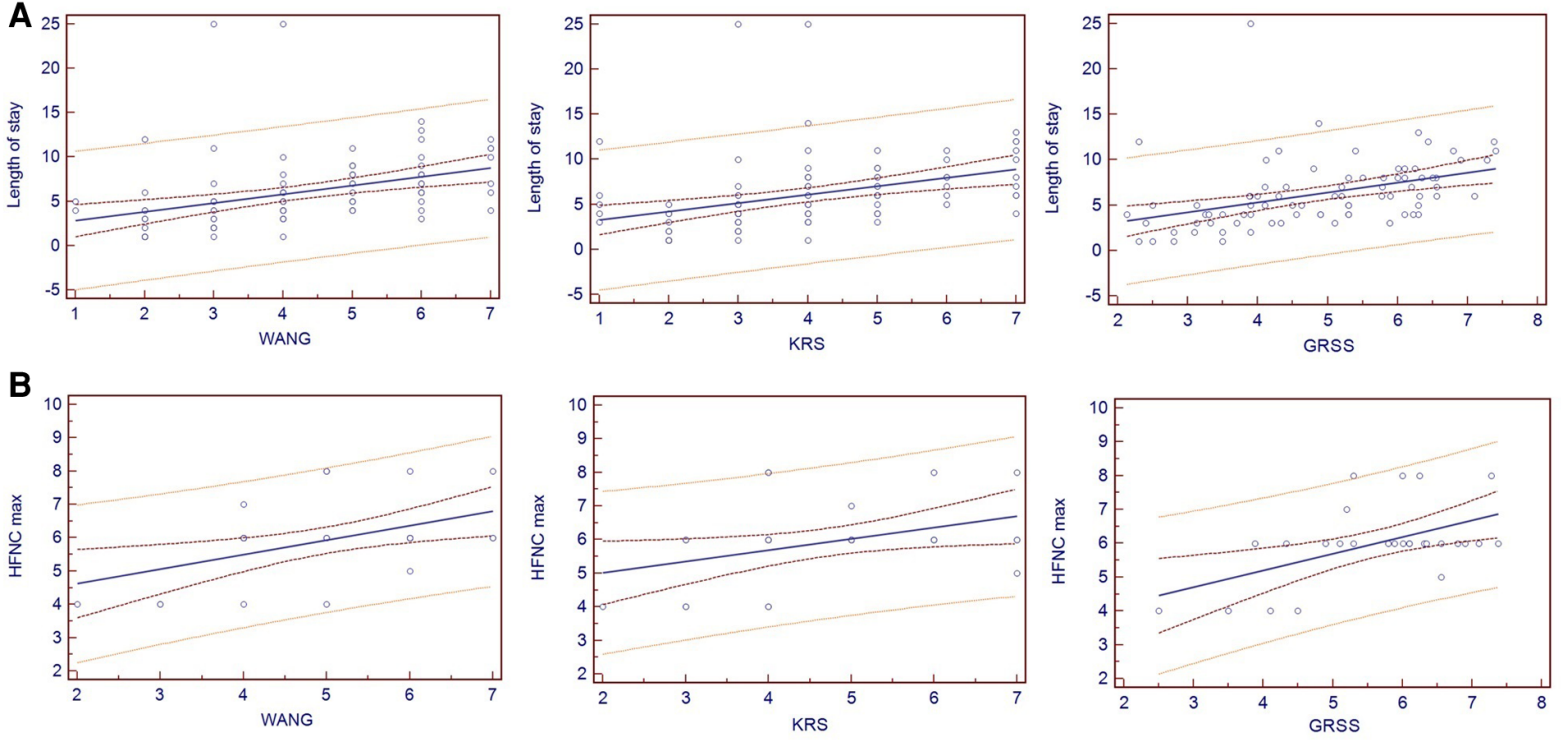
Spearman's correlation of the three different scores with the length of stay (Figure 2A) and the maximum flow used with high-flow nasal cannulas (Figure 2B).

Despite a low value for r^2^, all three scores were significantly correlated with the length of stay: WBSS with an r^2^ of 0.139 (*p* < 0.001), KRS with an r^2^ of 0.137 (*p* < 0.001), and GRSS with an r^2^ of 0.170 (*p* < 0.001) (Figure 2A).

All the scores significantly correlated with the maximum flow (minimum: 4 L/min, maximum: 8 L/min) used with high-flow nasal cannulas: WBSS with an r^2^ of 0.229 (*p* = 0.009), KRS with an r^2^ of 0.155 (*p* = 0.034) and GRSS with an r^2^ of 0.277 (*p* = 0.005) (Figure 2B). Infants who required CPAP had significantly higher WBSS and KRS scores (*p* < 0.001 and *p* < 0.001, respectively) (Table 6). The three infants who required mechanical ventilation had a median WBSS of 6.00 (IQR 5.00–6.50), a KRS of 7.00 (IQR 5.00–7.00), and a GRSS of 7.38 (IQR 5.59–7.39).

**Table 6:** Comparison of three bronchiolitis scores (WBSS, KRS, GRSS) between infants who required nasal continuous positive airway pressure (nCPAP) and infants managed only with high-flow nasal cannula (HFNC).

	nCPAP (*n* = 12)	HFNC (*n* = 59)	*p*-value
**Wang respiratory score (WBSS)**	6.00 (IQR 5.75–7.00)	4.00 (IQR 4.00–6.00)	<0.001
**Kristjansson respiratory score (KRS)**	7.00 (IQR 5.75–7.00)	4.00 (IQR 3.00–5.00)	<0.001
**Global Respiratory Severity Score (GRSS) [only for RSV infants (12 in the nCPAP group and 53 in the HFNC group)]**	6.53 (IQR 6.29–6.74)	5.10 (IQR 4.18–5.93)	0.063

## Discussion

This study is the first to compare the accuracy of three different severity scores in predicting the need for respiratory support and length of stay in neonates and small infants with bronchiolitis.

Criteria to hospitalize infants with bronchiolitis are hypoxia, moderate to severe respiratory distress, dehydration, or apnea; other criteria to be considered are comorbidities (such as prematurity, bronchopulmonary dysplasia, or congenital heart diseases) and unfavorable social and environmental factors (17). In a study evaluating infants with moderate to severe bronchiolitis, the saturation level on admission was the most critical predictor of hospitalization and the only one correlated with a longer hospital stay (18). Rodriguez-Martinez et al. systematically evaluated 32 instruments to evaluate the severity of bronchiolitis in different clinical settings: upon analyzing their content, respiratory rate turned out to be the most frequently used item (in 26/32, 81.3% of the instruments), followed by wheezing (in 25/32, 78.1% of the tools). They concluded that there is an urgent need to develop new instruments and better validate them (8). Furthermore, previous studies enrolled infants up to 24 months of life, where we know that patients younger than three months are at particular risk for severe bronchiolitis and frequently require prolonged hospitalization and intensive care. Therefore the need for a tool to help clinicians in the assessment of a neonate or infant with bronchiolitis, on admission and during hospitalization and to guide the respiratory support escalation is very high.

Our data support the hypothesis that all three scores (WBSS, KRS, and GRSS), evaluated on admission, can identify those patients who need respiratory support. The optimal cut-off for the WBSS, the KRS, and the GRSS were more than 3, 3, and 3.8, respectively, with an AUC of 0.903, 0.904, and 0.945. Furthermore, higher scores (WBSS of 6.00, KRS of 7.00, and a GRSS of 7.38) were founded in those patients requiring ICU admission for mechanical ventilation.

Consistently with results from another study (19), we found that a younger age at presentation is associated with more severe respiratory disease. At the same time, sex and weight on admission do not seem to predict our population's severity of bronchiolitis in our population.

All three scores were significantly correlated with the length of stay. As expected, the length of stay was longer for those patients receiving respiratory support, the same who presented with higher scores on admission.

Neonates and infants with RSV-bronchiolitis have a more severe clinical picture documented by significantly higher WBSS and KRS scores rather than patients infected with other viruses.

This study has two main limitations: first, the scores were evaluated retrospectively, analyzing medical records in a single center; second, no score has been specifically designed for the neonatal population (the only one that considers age between the items is the GRSS). Furthermore, in our department, HFNC was always used as first-line treatment in infants who needed oxygen therapy, considering the lower treatment failure in the group receiving high-flow oxygen therapy in a multicenter randomized controlled trial by Franklin et al. (14). This could have influenced results in infants with a milder disease who probably needed only standard oxygen therapy.

Furthermore, all scoring systems have a mix of objective and subjective data, which can create a bias in the evaluation. A good scoring system should consider only simple and well-categorized items that objectively measure respiratory status. In a world where an application for everything exists, a simple tool that all clinicians could use on their smartphone would be helpful and practical for use in daily clinical practice, without complicated calculations to do.

Finally, we noted that values > 3 for WBSS, > 3 for KRS, and >3.8 for GRSS discriminate with reasonable accuracy for neonates and infants who require ventilatory support. Further studies are needed to confirm our findings in a multicenter prospective study. These scoring tools may help design future clinical trials of treatments for bronchiolitis to have similar groups of patients to whom potential therapies can be applied. They also could be incorporated into protocols for escalation of respiratory support (HFNC, nCPAP, or other non-invasive supports).

## Conclusion

In neonates and infants younger than three months hospitalized with bronchiolitis, the WBSS, KRS, and GRSS clinical scores assessed on admission accurately discriminate patients with the most severe infections and correlate with the length of hospital stay. The GRSS score, which considers the patient's age, seems to have better sensitivity in neonates and small infants than other scores.

## Data Availability

The raw data supporting the conclusions of this article will be made available by the authors, without undue reservation.
